# Social Factors and Leukocyte DNA Methylation of Repetitive Sequences: The Multi-Ethnic Study of Atherosclerosis

**DOI:** 10.1371/journal.pone.0054018

**Published:** 2013-01-08

**Authors:** Malavika A. Subramanyam, Ana V. Diez-Roux, J. Richard Pilsner, Eduardo Villamor, Kathleen M. Donohue, Yongmei Liu, Nancy S. Jenny

**Affiliations:** 1 Social Epidemiology, Indian Institute of Technology Gandhinagar, Ahmedabad, Gujarat, India; 2 Department of Epidemiology, University of Michigan, Ann Arbor, Michigan, United States of America; 3 Division of Environmental Health Sciences, University of Massachusetts, Amherst, Massachusetts, United States of America; 4 Columbia University Medical Center, Columbia University, New York, New York, United States of America; 5 School of Medicine, Wake Forest University, Winston-Salem, North Carolina, United States of America; 6 Laboratory for Clinical Biochemistry Research, University of Vermont, Colchester, Vermont, United States of America; Geisel School of Medicine at Dartmouth, United States of America

## Abstract

Epigenetic changes are a potential mechanism contributing to race/ethnic and socioeconomic disparities in health. However, there is scant evidence of the race/ethnic and socioeconomic patterning of epigenetic marks. We used data from the Multi-Ethnic Study of Atherosclerosis Stress Study (N = 988) to describe age- and gender- independent associations of race/ethnicity and socioeconomic status (SES) with methylation of Alu and LINE-1 repetitive elements in leukocyte DNA. Mean Alu and Line 1 methylation in the full sample were 24% and 81% respectively. In multivariable linear regression models, African-Americans had 0.27% (p<0.01) and Hispanics 0.20% (p<0.05) lower Alu methylation than whites. In contrast, African-Americans had 0.41% (p<0.01) and Hispanics 0.39% (p<0.01) higher LINE-1 methylation than whites. These associations remained after adjustment for SES. In addition, a one standard deviation higher wealth was associated with 0.09% (p<0.01) higher Alu and 0.15% (p<0.01) lower LINE-1 methylation in age- and gender- adjusted models. Additional adjustment for race/ethnicity did not alter this pattern. No associations were observed with income, education or childhood SES. Our findings, from a large community-based sample, suggest that DNA methylation is socially patterned. Future research, including studies of gene-specific methylation, is needed to understand better the opposing associations of Alu and LINE-1 methylation with race/ethnicity and wealth as well as the extent to which small methylation changes in these sequences may influence disparities in health.

## Introduction

Health disparities by race/ethnicity [1], [2] and socioeconomic status (SES) [3] have been repeatedly documented. Social and physical exposures linked to race/ethnicity and SES could exert biologic effects through changes in gene expression [4], [5]. Epigenetic markers have been increasingly incorporated into epidemiologic studies of outcomes ranging from ischemic heart disease to various cancers [6], [7]. However, there is limited evidence on the extent to which epigenetic marks are systematically patterned by the race/ethnic and socioeconomic characteristics for which a range of health disparities are observed.

DNA methylation is one of the most frequently studied epigenetic changes [8], [9]. Studies suggest that DNA methylation is modified by environmental factors and that these changes in DNA methylation levels occur over the lifecourse [10], [11]. Global DNA methylation refers to methylation levels in the whole genome and is frequently estimated using surrogate measures such as methylation in Alu and LINE-1 repetitive elements, which represent approximately 30% of the genome [8], [9]. Global DNA hypomethylation is associated with exposures such as lead [9] and ambient black carbon [12] as well as outcomes such as ischemic heart disease and stroke among others, [6] suggesting that environmental exposures lead to epigenetic changes that influence disease outcomes.

While the need for investigating the association of global DNA methylation with demographic and lifestyle factors has been highlighted, [8] to date few studies have examined these questions. One study pooled data from 1465 participants in Italy, Poland and the United States (U.S.) and found that age and alcohol consumption were inversely associated with Alu methylation and that males were more likely to have lower Alu methylation but higher LINE-1 methylation than females [8]. Of the few studies that have examined race/ethnic and socioeconomic differences in global DNA methylation, [13], [14] one study of 85 women in New York found that African-Americans were more likely to have lower DNA methylation than whites. Low SES was also associated with lower global DNA methylation, although it was not statistically significant [13]. In contrast, a study of 28 women found no differences in global DNA methylation between Whites and African-Americans [14]. A recent study of 239 participants of the Glasgow psychological, social and biological determinants of ill health (pSoBid) cohort found that global DNA methylation in peripheral leukocytes (measured using antibody binding to 5-methylcytosine) was lower among the deprived and the manual social class, compared to the affluent and the non-manual class respectively [15]. A genome-wide methylation analysis of blood DNA from 40 adult men in the 1958 British Birth Cohort Study found that childhood SES was associated with methylation levels of 1252 gene promoters (666 positive and 586 inverse associations) while adult SES was associated with methylation levels of 545 promoters (336 positive and 209 inverse associations) [16]. To our knowledge no large community-based study has examined the race/ethnic and socioeconomic patterns in DNA methylation in the U.S. context.

We used data from the Stress Ancillary Study of the Multi Ethnic Study of Atherosclerosis (MESA) to investigate the association of DNA methylation of Alu and LINE-1 repetitive sequences with race/ethnicity and socioeconomic status in a large population sample. Specifically, we assessed the age- and gender -independent association of Alu and LINE-1 DNA methylation with race/ethnicity, income, wealth, education, and childhood SES. We also investigated whether the associations of Alu and LINE-1 methylation with race/ethnicity and socioeconomic factors varied by gender.

## Methods

### Data

We used data from the MESA Stress Study, an ancillary study to the MESA, which was funded by the National Heart Lung and Blood Institute to investigate risk factors of subclinical cardiovascular disease (CVD) and its progression to clinical disease. At baseline (2000–2002) 6814 participants, aged 44 to 84 years and without clinical CVD, were recruited to MESA from six sites across the U.S using diverse population-based approaches [17].

The MESA Stress Study is a subsample of MESA participants recruited at the New York and Los Angeles study sites (n = 1002). Participants were enrolled in 2004–2006 in the order in which they attended the MESA follow-up exams until about 500 participants were enrolled per site. MESA Stress participants were similar to the large MESA cohort with the exceptions that they had fewer individuals aged 75–84 (12.1% vs. 18.2%), more men (47.6% vs. 44.7%) and more college educated participants (29.7% vs. 23.9%).

The Multi-Ethnic Study of Atherosclerosis was approved by institutional review boards at the six field centers: Columbia University, New York; Johns Hopkins University, Baltimore; Northwestern University, Chicago; UCLA, Los Angeles; University of Minnesota, twin Cities; Wake Forest University, Winston-Salem. All participants provided written informed consent.

### Social factors

Race/ethnicity reported by participants was categorized as white, African-American and Hispanic. Race/ethnicity was considered a social factor because of evidence showing strong patterning of various social exposures by race/ethnicity in the Unites States [2].

The following self-reported measures of SES were investigated: income, wealth, education, and childhood SES (as proxied by education of the participant's father).

Using data on total family income, a 13 category variable ranging from <$5,000 to >$100,000, a continuous income variable was created by assigning the mid-point of each income category to the participant (those who reported total family income of <$5,000 were assigned a value of $2,500 and those with >$100,000 were assigned $112,500 based on the U.S. income distribution.) To account for family size, this continuous income variable was divided by the number of family members and standardized by subtracting each individual's value from the study sample mean and dividing by the standard deviation.

A 5-point wealth index used in prior MESA work, [18] ranging from 0 to 4, was calculated by giving 1 point to ownership of each of the following assets: car (one or more), a home (own/paying mortgage), land, investments (for e.g. stocks, bonds, mutual funds) and summing the points. Similar to income, this was then converted into a *z* score for use in models.

Education was operationalized as a *z* score of the number of years of schooling. Following the approach used in prior work [19]–[23] participant's childhood SES was measured using his or her father's education categorized into six levels (no schooling, less than high school, high school, some college, college degree, and graduate degree). This variable was transformed into a six point continuous score and *z* scored for analyses.

Other covariates included in the analysis were age (45–54, 55–64, 65–74, and 75–84) and gender. Data on wealth was from the 3^rd^ examination of the MESA cohort (2004–2005) while all other variables were measured at baseline.

### Alu and LINE-1 DNA methylation

DNA methylation is a distinct marker of epigenetic changes that regulate several biological processes. Repetitive elements, which represent about 30% of the human genome, are estimated to be the site of more than 1/3 of DNA methylation [8]. We used methylation levels of Alu and LINE-1 repetitive elements as markers of global DNA methylation levels. Description of collection and storage of blood samples has been reported previously [17]. DNA samples from the leukocytes in the baseline blood sample (500 ng at 20 ng/μl) were bisulfite-treated using the EZ-96 DNA Methylation Kit (Zymo Research, Orange, CA). Bisulfite conversion of DNA changes unmethylated cytosine to uracil and subsequently to thymidine after PCR whereas methylated cytosines are protected from bisulfite conversion, resulting in methylation-dependent differences in DNA sequences. LINE-1 and Alu methylation were measured by pyrosequencing using PCR primers and running conditions previously described [9]. Sample controls included human genomic DNA that had undergone whole-genome amplification to remove CpG methylation for a 0% methylated control and a human methylated standard (Zymo Research, Orange CA) for a 100% methylated control. Samples were sequenced on a PSQ HS96 Pyrosequencing System. The % methylation (methylated/unmethylated) for each CpG target region was quantified using the Pyro Q-CpG Software. This software assigns quality scores for each measurement and internal quality controls to assess the efficiency of bisulfite conversion. The interassay coefficients of variation for LINE-1 and Alu were 2.10% and 5.73%, respectively. Data on Alu (3 sites per participant) and LINE-1 (4 sites per participant) were available for 987 and 961 participants respectively. For both Alu and LINE-1, we used the percentage of CpG sites that were methylated as the outcome variables.

### Statistical Analysis

We described the distribution of key predictors, covariates, and outcomes. We also used analysis of variance, and when appropriate tests of linear trend, to investigate whether the mean (standard deviation) of methylation differed across categories of covariates.

Regression models were used to estimate associations of social factors with methylation after adjustment for covariates. Linear mixed models were used to account for the multiple sites measured within individuals. Models accounted for within subject correlations and included a random coefficient for site to account for the high between-site variability in average methylation (Table S1). Estimates of associations were derived using an unstructured covariance structure and robust standard errors.

To examine differences by race/ethnicity we estimated age- and gender- adjusted mean differences by race/ethnicity. Because of the high levels of race/ethnic inequalities in SES, [2] we further adjusted for SES while estimating the association of race/ethnicity with DNA methylation. We also investigated differences by SES by estimating mean age- and gender- adjusted differences before and after adjusting for race/ethnicity, adjustment for race/ethnicity was deemed necessary because of the strong association of SES and race/ethnicity. Because we were interested in the unique association of each SES indicator with methylation and also due to the relatively high correlation among the SES indicators, separate models were fit for each SES indicator.

In order to assess the interaction of gender with race/ethnicity and SES we included appropriate interaction terms in the models.

## Results

The average age of the 988 participants in whom either Alu or LINE-1 methylation measures were available was 61 years (SD = 9.9) and a majority were female (52%). Alu methylation level was about 24% on average (SD = 1.15) and LINE-1 was 81% (SD = 1.66). The correlation of Alu and LINE-1 methylation was 0.10 (p<0.01). The distributions of race/ethnicity and SES were approximately similar in men and women, although women tended to have less income than men (not shown). In general African-Americans and Hispanics had lower SES (for both adult and childhood measures) than whites (not shown). Sample distributions were very similar for the Alu (n = 987) and Line-1 (n = 961) subsamples (Table 1).

**Table 1:** Sample characteristics (%) and mean (SD) of Alu and LINE-1 methylation across categories of age, race/ethnicity, income, wealth, education, and childhood SES in the study sample.

		Alu^†^		LINE-1^†^	
		N = 987	Mean (SD)	N = 961	Mean (SD)
**Gender**	Male	47.5	24.4 (1.1)	47.6	81.1 (1.6)***
	Female	52.5	24.4 (1.2)	52.5	80.4 (1.7)
**Age**	45 to 64	30.2	24.4 (1.1)	30.2	80.6 (1.7)
	55 to 64	27.5	24.5 (1.2)	27.4	80.7 (1.7)
	65 to 74	30.3	24.3 (1.1)	30.4	80.9 (1.6)
	75 to 84	12.0	24.8 (1.1)	12.0	80.9 (1.8)
**Race/ethnicity**	White	18.7	24.7 (1.3)**	18.7	80.4 (1.9)**
	African American	28.1	24.4 (1.1)	28.2	80.9 (1.5)
	Hispanic	53.2	24.4 (1.1)	53.1	80.8 (1.6)
**Income**	<$25,000	39.4	24.4 (1.1)	38.7	80.8 (1.7)
	$25-49,999	34.3	24.4 (1.1)	35.0	80.7 (1.6)
	>$49,999	26.3	24.6 (1.1)	26.3	80.7 (1.7)
**Wealth**	0 assets	18.8	24.3 (1.2)**	18.7	81.0 (1.6)**
	1 asset	26.2	24.3 (1.2)	26.5	80.8 (1.7)
	2 assets	25.4	24.4 (1.1)	25.2	80.7 (1.5)
	3 assets	17.8	24.7 (1.2)	17.6	80.8 (1.7)
	4 assets	11.8	24.7 (1.2)	12.0	80.3 (1.8)
**Education**	Less than high school	27.1	24.3 (1.2)	26.7	80.7 (1.7)
	High school	20.3	24.5 (1.1)	20.4	81.0 (1.7)
	Some college	29.7	24.5 (1.2)	29.9	80.6 (1.5)
	College degree or more	22.9	24.4 (1.2)	23.0	80.7 (1.8)
**Childhood SES** ‡	Low	64.7	24.4 (1.1)	64.6	80.8 (1.6)
	Medium	18.6	24.4 (1.1)	18.3	80.6 (1.8)
	High	16.7	24.6 (1.2)	17.1	80.7 (1.8)

‡ Childhood SES: Low  =  less than high school, medium  =  high school degree, high  =  some college or more. **^†^**Missing in Alu and LINE-1 samples: Income (14), wealth (3), childhood SES (39 in Alu, 37 in LINE-1) *  =  p<0.05, **  =  p<0.01, ***  =  p<0.001.

Men had significantly higher LINE-1 methylation than women (81.1 vs. 80.4, p<0.0001) (Table 1). African-Americans and Hispanics had lower Alu but higher LINE-1 methylation compared to whites (p value for both tests <0.01). Alu methylation was higher, and LINE-1 methylation lower, at higher levels of wealth (p value from both tests of trend <0.01).

The mean differences in repetitive sequence DNA methylation levels by age, gender and race/ethnicity before and after adjustment for SES indicators are shown in Table 2. LINE-1 was positively associated with age. In comparison to the youngest group, participants aged 65 to 74 had a higher methylation (mean difference 0.31% ±0.13; p<0.05) as did those aged 75 years or more (mean difference 0.48% ±0.17; p<0.01). In contrast, Alu methylation did not show a similar pattern although compared to the youngest group, participants who were 75 years of age and over had higher methylation (0.26% ±0.11; p<0.05). Men consistently had higher levels of methylation than women; however, gender differences in LINE-1 methylation were much larger than in Alu and were statistically significant (0.62% ±0.10; p<0.001).

**Table 2:** Mean differences (SE) in DNA methylation levels associated with age, gender and race.

	Alu		LINE-1	
	Age, gender and race	Additionally adjusted for all SES indicators†	Age, gender and race	Additionally adjusted for all SES indicators†
**Age**				
45 to 54	Ref	Ref	Ref	Ref
55 to 64	−0.01 (0.08)	−0.05 (0.08)	0.04 (0.13)	0.06 (0.13)
65 to 74	−0.15 (0.08)	−0.08 (0.08)	0.28 (0.12)*	0.31 (0.13)*
75 to 84	0.24 (0.11)*	0.26 (0.11)*	0.43 (0.16)**	0.48 (0.17)**
**Gender**				
Male	0.07 (0.06)	0.07 (0.06)	0.60 (0.10)***	0.62 (0.10)***
Female	Ref	Ref	Ref	Ref
**Race category**				
White	Ref	Ref	Ref	Ref
African American	−0.27 (0.09)**	−0.29 (0.10)**	0.41 (0.14)**	0.38 (0.16)*
Hispanic	−0.20 (0.08)*	−0.23 (0.11)*	0.39 (0.13)**	0.39 (0.17)*

† SES indicators adjusted were: income, wealth, education, childhood SES.

* =  p<0.05, **  =  p<0.01, ***  =  p<0.001.

We found statistically significant race/ethnic differentials in Alu and LINE-1 methylation; however, the associations were in opposing directions. African-American and Hispanic participants had significantly lower Alu methylation than whites, with African-Americans having a 0.27% (±0.09, p<0.01) and Hispanics a 0.20% (±0.08, p<0.05) lower methylation than whites in age and gender adjusted models. These patterns remained largely unchanged after further adjustment for SES. In contrast, African-Americans and Hispanics had higher LINE-1 methylation than whites. African-Americans had, on average, 0.41% (±0.14, p<0.01) higher LINE-1 methylation than whites in age and gender adjusted models. Hispanics had a similar 0.39% (±0.13, p<0.01) higher LINE-1 methylation compared to whites. This pattern remained after adjustment for SES indicators.

Table 3 shows associations of each SES indicator with methylation levels adjusting for age and gender, and further adjusting for race/ethnicity. Each SES indicator was examined separately. Wealth was positively associated with Alu and inversely associated with LINE-1. On average, 1 SD higher wealth was associated with 0.09% (±0.03, p<0.01) higher Alu and 0.15% (±0.05, p<0.01) lower LINE-1 methylation in age- and gender- adjusted models. The estimates did not change after adjusting for race/ethnicity. The other SES indicators-income, education and childhood SES-were not associated with DNA methylation.

**Table 3:** Mean differences (SE) in DNA methylation levels associated with income, wealth, education, and childhood SES.

	Alu		LINE-1	
	Adjusted for age and gender	Adjusted for age, gender and race	Adjusted for age and gender	Adjusted for age, gender and race
Income	0.004 (0.031)	−0.037 (0.035)	−0.04 (0.05)	0.02 (0.06)
Wealth	0.09 (0.03)**	0.08 (0.03)*	−0.15 (0.05)**	−0.12 (0.05)*
Education	0.04 (0.03)	0.01 (0.04)	−0.03 (0.05)	0.04 (0.06)
Childhood SES	0.06 (0.03)	0.02 (0.04)	−0.04 (0.05)	0.04 (0.06)

* =  p<0.05, **  =  p<0.01, ***  =  p<0.001.

There was no evidence of an interaction of race/ethnicity with gender. Among the SES indicators, there was a statistically significant interaction of education with gender (p for interaction  = 0.02 for both Alu and LINE-1). In gender-stratified models adjusted for age and race/ethnicity, education was positively associated with methylation in men (Alu  = 0.12%, ±0.05; p = 0.01 and LINE-1  = 0.22% ±0.08; p<0.01), while the associations were inverse and not statistically significant in women (Alu  = −0.07%±0.05 and LINE-1 = −0.12±0.08). We did not find statistically significant interactions of other SES indicators with gender.

## Discussion

We observed a pattern of higher Alu and lower LINE-1 methylation among socially advantaged versus disadvantaged groups in a large population-based multi-ethnic sample of adults aged 45–84 from New York and Los Angeles.

Our finding that whites had higher Alu and lower LINE-1 methylation compared to African-Americans and Hispanics adds to the small number of studies that have investigated race/ethnic differences in global DNA methylation. Our result is in contrast to the finding of lower LINE-1 methylation among African-Americans and Hispanics versus whites that was reported in a Texas study of 161 participants aged >45 [24]. While a New York study comparing global DNA methylation in 85 women found that 52% of whites (versus 24% African-American and 71% Hispanic) had DNA methylation levels above the median of the distribution, the study did not use Alu or LINE-1 methylation to measure genomic DNA methylation [13].

We found that wealth was positively associated with Alu and inversely associated with LINE-1 methylation. Two prior studies have investigated socioeconomic differences in DNA methylation. Among 85 women from a New York City cohort [13] there was no evidence that leukocyte DNA methylation was associated with family SES. In contrast, global DNA methylation was positively associated with wealth (deprived vs. affluent) and social class (manual vs. non-manual) in 239 participants from the Glasgow pSoBid cohort, although there was no evidence of an association with income or education [15]. Notably these two studies used [^3^H]-methyl acceptance assay and antibody binding to 5-methylcytosine to measure genomic DNA methylation and not Alu or LINE-1 methylation. Our finding of a positive association of wealth with Alu methylation corresponds with the results of the pSoBid study although we found evidence of a negative association of wealth with LINE-1.

Given at least some prior work linking social [13], behavioral [8] and environmental exposures [9], [12] as well as disease outcomes [6] patterned by SES and race/ethnicity to hypomethylation, we expected to see less methylation in the more socially disadvantaged groups. This was true for Alu but not for LINE-1 methylation. Not all adverse health factors have been linked to hypomethylation. For example, global DNA hypermethylation has been linked to elevated leucocyte count and inflammatory markers, and CVD [25]. Also, adverse environmental exposures have been linked to hypothemylation at ALU but not at LINE-1 sites [26], [27]. Additional work is needed to replicate these patterns in other samples.

Notably, wealth was the only socioeconomic indicator that was consistently associated with DNA methylation. Among the same participants, we found previously that wealth was the most consistent socioeconomic predictor of cortisol levels [18]. The relevance of the wealth measure may be related to the relatively older age of the sample, given that wealth is an especially valid SES measure in older populations [28], [29]. Also, our sample had a large representation of African-Americans and Hispanics, including recent immigrants. Income and education have limitations in characterizing SES in these populations [3]. This may explain the absence of associations of income and education with methylation in this sample (with the possible exception of education among men). The finding that wealth was a stronger predictor of DNA methylation than education or income is consistent with recent findings of the pSoBid study [15]. Our measure of childhood SES was limited in that it focused on parental education and had limited variability in our sample (65% had less than high school education). Given the possible relevance of early life exposures to epigenetic processes [30] additional work with better measures of childhood SES is needed.

A number of plausible mechanisms exist through which social circumstances could affect levels of methylation. Social experiences early in life have been shown to be related to epigenetic changes including methylation [16], [30]–[32]. Diet is known to be patterned by race/ethnicity [33], [34] and SES [35], [36] and dietary factors have in turn been linked to global DNA methylation [37], [38]. Other socially patterned behaviors, such as physical activity [39] and alcohol intake [40] have also been linked to methylation. A number of environmental exposures known to be patterned by SES and race/ethnicity [41], [42] have been linked to methylation, including lead, [9], [43] arsenic, [44] benzene, [45] persistent organic pollutants including organochlorine pesticides, [26] and various pollutants in the air [46]–[48].

We investigated leukocyte DNA methylation of Alu and LINE-1, two different repetitive sequences frequently used in epidemiologic studies. Although both are thought to act as a surrogate for global DNA methylation, the weak correlation of Alu with LINE-1 methylation in normal tissues [49] and the difference in their associations with cellular and environmental exposures [26], [45] have been previously reported. This suggests that both measures may interrogate different cellular processes. Alu and LINE-1 elements use different internal RNA polymerase promoters and Alu elements have no coding capacity [50]. Due to these differences, the CpG sites of Alu and LINE-1 may be under different selective pressures [51]. Lastly, these elements are highly polymorphic, [52], [53] for example, the lower methylation values for Alu versus LINE-1 have been linked to high levels of polymorphisms within CpG dinucleotides of the consensus sequence [53]. All these factors could lead to differential associations of these markers with sociodemographic factors.

Measurement error in methylation could also have affected our findings [51]. Deviations from the consensus sequence created by polymorphisms and/or deletions may lead to stalls in pyrosequencing or misincorporation of nucleotides, thereby affecting the quantitative reading of the methylation values [54]. Higher annealing temperatures were reported to produce larger differences in LINE-1 methylation between males and females [54]. It is not known if differences in annealing temperature may similarly influence the magnitudes of association between other exposures and Alu methylation.

A limitation of our study is that we analyzed DNA from leukocytes which may not represent the tissue most affected by the social antecedents we were interested in. Differences in leukocyte subtype count have been linked to methylation [8], [55] and to gender and race/ethnicity [56] and could confound our findings. Thus, we cannot completely rule out that our findings may be due to shifts in leukocyte subtype counts. Despite this limitation, results from recent studies that have analyzed leukocyte DNA methylation have been informative [10], [57]. For instance, DNA methylation of the glucocorticoid receptor in leukocytes of infants was related to cortisol responsivity to stress [57]. There is also some evidence that concordance of tissue-specific methylation patterns may be greater than previously thought [58], [59]. Our ability to examine childhood SES was limited by the measures available in the MESA dataset. Future studies will need to examine more complete comprehensive measures of childhood SES including those related to parental occupation and household resources such as housing and wealth. The cross-sectional design precludes drawing any conclusions about the temporal order of SES and DNA methylation. The exclusion of adults with a history of clinical CVD in the MESA Study mean that our analyses are restricted to a healthier subsample which may have affected our ability to detect associations.

Strengths of our study include the large and diverse population sample and the use of widely-used measures of adult and childhood SES. Further examinations of epigenetic changes linked to race/ethnicity and socioeconomic factors using more specific markers of DNA methylation may provide important clues on how social disadvantages are translated into biological structure and function.

## Supporting Information

Table S1 **Mean (SD) of Alu and LINE-1 methylation at each site and the between-site Pearson correlations in the MESA Stress Study sample.**
(DOCX)
