# Supplementary material for: Social Factors and Leukocyte DNA Methylation of Repetitive Sequences: The Multi-Ethnic Study of Atherosclerosis
Source: PLoS One. 2013 Jan 8;8(1):e54018. doi: 10.1371/journal.pone.0054018 (PMC3539988; doi:10.1371/journal.pone.0054018)
Supplement: Table S1 — Mean (SD) of Alu and LINE-1 methylation at each site and the between-site Pearson correlations in the MESA Stress Study sample. (DOCX) [file pone.0054018.s001.docx]

**Table S1** Mean (SD) of Alu and LINE-1 methylation at each site and the between-site Pearson correlations in the MESA Stress Study sample.

|  | **All** | | **Male** | | **Female** | |
| --- | --- | --- | --- | --- | --- | --- |
| **Mean (SD)** | **ALU (N = 987)** | **LINE-1 (N = 961)** | **ALU (N = 469)** | **LINE-1 (N = 457)** | **ALU (N = 518)** | **LINE-1 (N = 504)** |
|  |  |  |  |  |  |  |
| Site 1 | 31.82 (1.55) | 79.92 (2.81) | 31.79 (1.45) | 80.14 (2.81) | 31.85 (1.64) | 79.72 (2.80) |
|  |  |  |  |  |  |  |
| Site 2 | 26.40 (1.61) | 81.91 (1.62) | 26.41 (1.58) | 82.23 (1.62) | 26.39 (1.64) | 81.63 (1.56) |
|  |  |  |  |  |  |  |
| Site 3 | 15.07 (1.03) | 76.94 (2.48) | 15.13 (0.87) | 77.34 (2.55) | 15.01 (1.16) | 76.59 (2.35) |
|  |  |  |  |  |  |  |
| Site 4 |  | 84.14 (2.48) |  | 84.52 (2.46) |  | 83.79 (2.46) |
|  |  |  |  |  |  |  |
| **Between-site correlations** | | |  |  |  |  |
| Sites 1,2 | 0.59*** | 0.28*** | 0.61*** | 0.22*** | 0.57*** | 0.32*** |
|  |  |  |  |  |  |  |
| Sites 1,3 | 0.33*** | 0.09** | 0.39*** | 0.02 | 0.30*** | 0.14** |
|  |  |  |  |  |  |  |
| Sites 2,3 | 0.56*** | 0.51*** | 0.58*** | 0.42*** | 0.55*** | 0.58*** |
|  |  |  |  |  |  |  |
| Sites 1,4 |  | 0.44*** |  | 0.43*** |  | 0.45*** |
|  |  |  |  |  |  |  |
| Sites 2,4 |  | 0.49*** |  | 0.43*** |  | 0.52*** |
|  |  |  |  |  |  |  |
| Sites 3,4 |  | 0.26*** |  | 0.23*** |  | 0.26* |
| * = p< 0.05, ** = p< 0.01, *** = p< 0.001 | | |  |  |  |  |
